# Non-Thermal Atmospheric Pressure Bio-Compatible Plasma Stimulates Apoptosis via p38/MAPK Mechanism in U87 Malignant Glioblastoma

**DOI:** 10.3390/cancers12010245

**Published:** 2020-01-19

**Authors:** Mahmuda Akter, Anshika Jangra, Seung Ah Choi, Eun Ha Choi, Ihn Han

**Affiliations:** 1 Department of Plasma Bio-Display, Kwangwoon University, Seoul 01897, Korea; nipa21stfeb@gmail.com (M.A.); ehchoi@kw.ac.kr (E.H.C.); 2 Plasma Bioscience Research Center, Applied Plasma Medicine Center, Kwangwoon University, Seoul 01897, Korea; 3 Department of Neurosurgery, Seoul National University Hospital, Seoul National University College of Medicine, Seoul 01897, Korea; anshikajangra205@gmail.co (A.J.); aiipo@snu.ac.kr (S.A.C.); 4 Department of Electronic and Biological Physics, Kwangwoon University, Seoul 01897, Korea

**Keywords:** nonthermal biocompatible plasma, soft jet plasma, reactive oxygen and nitrogen species, human glioblastoma, p38/MAPK pathway

## Abstract

Nonthermal plasma is a promising novel therapy for the alteration of biological and clinical functions of cells and tissues, including apoptosis and inhibition of tumor progression. This therapy generates reactive oxygen and nitrogen species (RONS), which play a major role in anticancer effects. Previous research has verified that plasma jets can selectively induce apoptosis in various cancer cells, suggesting that it could be a potentially effective novel therapy in combination with or as an alternative to conventional therapeutic methods. In this study, we determined the effects of nonthermal air soft plasma jets on a U87 MG brain cancer cell line, including the dose- and time-dependent effects and the physicochemical and biological correlation between the RONS cascade and p38/mitogen-activated protein kinase (MAPK) signaling pathway, which contribute to apoptosis. The results indicated that soft plasma jets efficiently inhibit cell proliferation and induce apoptosis in U87 MG cells but have minimal effects on astrocytes. These findings revealed that soft plasma jets produce a potent cytotoxic effect via the initiation of cell cycle arrest and apoptosis. The production of reactive oxygen species (ROS) in cells was tested, and an intracellular ROS scavenger, N-acetyl cysteine (NAC), was examined. Our results suggested that soft plasma jets could potentially be used as an effective approach for anticancer therapy.

## 1. Introduction

Glioblastoma astrocytoma is the most familiar and quickly progressing type of astrocytic brain tumor in adults, with a five-year survival rate of around ~4%. Glioblastoma is one of the most lethal types of brain cancer [1]. Due to its excessive resistance to conventional therapy, the mean survival time of patients is no more than 15 months [1,2]. Treatment for glioblastoma is inadequate; most chemotherapeutics are unable to cross the blood–brain barrier to reach tumor sites, and successfully removing all surrounding tumor cells by surgical resection is difficult [3].

Nonthermal plasma and its biomedical applications have recently become a major focus of research [4,5,6,7,8,9,10]. Plasma has been shown to induce apoptosis in various cancer cells [5,6,7]. Nonthermal atmospheric biocompatible plasma (NBP) was found to produce promising anticancer effects on glioblastoma cells in vitro [11] and in vivo [12]. Atmospheric pressure plasma jets can significantly destroy cancer cells without damaging healthy cells. Reactive oxygen species (ROS) and reactive nitrogen species (RNS) mostly appear in the human body and, at lower concentrations, mediate several biological actions, including platelet aggregation, vasodilation, apoptosis, and smooth muscle cell proliferation [13]. The creation of ROS could be used for the treatment of cancer [14]. NBP induces the production of intracellular ROS and activates DNA damage signaling, including p53 expression [15,16]. These reactive species have a key function in the induction of apoptosis in cancer cells specifically exposed to NBP [5,17,18,19] or indirectly treated by plasma-activated water or materials [20,21,22,23,24,25,26,27]. Nonthermal atmospheric pressure plasma jets (NBP-Js) have gained attention in the biomedical field [28,29,30,31,32]. NBP not only has the capacity to stimulate apoptosis in glioblastoma cells, but also has minimal cytotoxic effects on normal astrocytes [33,34]. Various studies have verified that reactive oxygen and nitrogen species (RONS) created by plasma can trigger mechanisms that lead to apoptosis, including cell signaling pathways involving c-Jun N-terminal kinases (JNK), p38, and p53 [35].

Thus, NBP-J has the potential for use in anticancer treatment, but determining mechanisms underlying its anticancer effects is necessary. In this study, we investigated the apoptotic effect of NBP-J, without damaging normal cells, and its capacity to alter oxidative stress pathways in human brain cancer cells. We identified molecular mechanisms triggered by plasma treatment, including molecular- and cell-cycle-related events that block cell cycle progression in cancer cells and signaling pathways responsible for the low survival and proliferation of glioblastoma cells treated with NBP-J.

## 2. Results

### 2.1. Physical Characteristics of the Soft Plasma Jet

**Figure 1:**
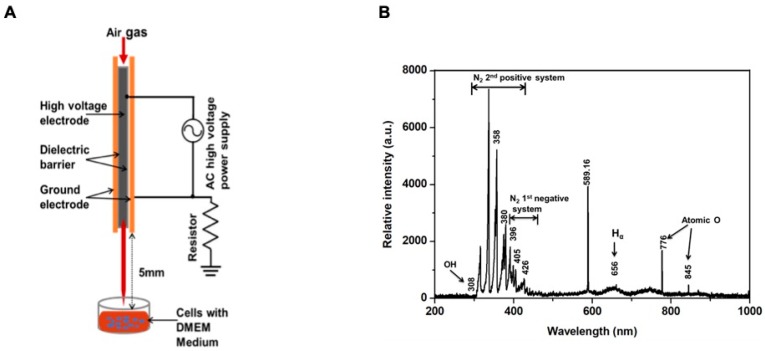
(**A**) Schematic overview of the experimental setup; (**B**) measurement of optical emission spectra (OES) of the soft plasma jet device with air gas flow.

Figure 1A provides a schematic diagram of a soft plasma jet device consisting of a high-voltage power supply, electrodes, and a dielectric barrier. We used a nonthermal plasma jet with air gas flow. The flow rate was 1 L/min, and a 1 mm diameter gas hole for open air was used for system operation. The discharge gap was maintained at 2 mm between the inner and outer electrodes. The gap between the surface of the Dulbecco’s modified Eagle medium (DMEM) and the tip of the plasma discharge device was fixed to 5 mm.

Figure 1B shows the optical emission spectra, indicating peak wavelengths. The emission lines shown in Figure 1B correspond to highly RNS or ROS that may produce biological effects. The discharge produced considerable UV radiation with an OH band transition at 308 nm, the second positive system of N_2_ from 314 to 388 nm, which included RNS, the first negative system N_2_ from 390 to 440 nm, hydrogen-α at 656 nm, and atomic oxygen lines at 777 and 844 nm. This ambient oxygen and energized nitrogen species may be included within the oxidation of different molecules by the deletion of electrons that could influence several biological processes.

### 2.2. Morphological Characteristics after Plasma Treatment

**Figure 2:**
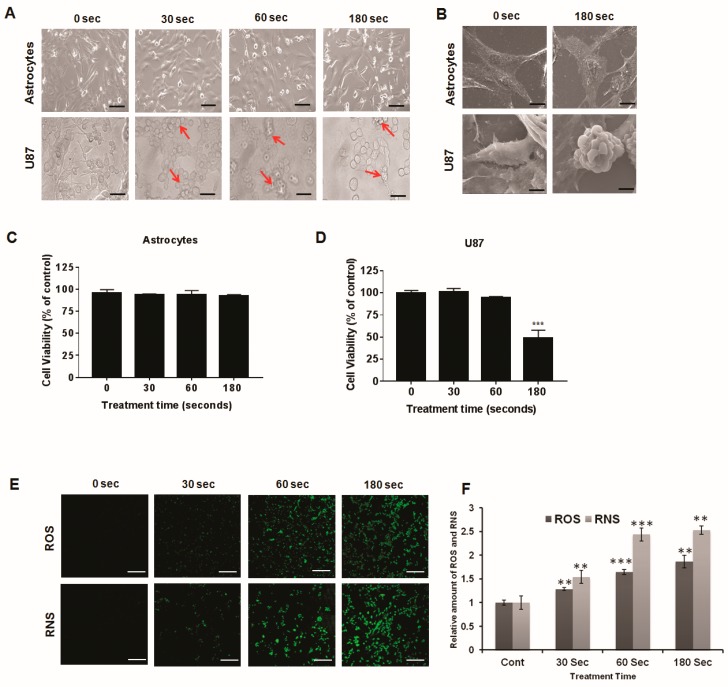
(**A**) Morphological changes in cell lines of astrocytes and U87 MG cells treated with plasma for 30, 60, and 180 s and viewed under a light microscope. Arrows indicate cell shrinkage and nuclear condensation due to apoptosis and the presence of apoptotic bodies. (**B**) SEM morphology of astrocytes and U87 MG cells. Effects of a soft plasma jet on cell viability of astrocytes (**C**) and U87 MG cells (**D**). (**E**) Confocal microcopy images of intracellular reactive oxygen species (ROS) and intracellular reactive nitrogen species (RNS) of U87 MG cells. (**F**) Relative amounts of ROS and RNS levels following soft plasma jet treatment of U87 MG cells. Scale bar = 100 μm. All values are presented as means ± SD of three independent experiments. ** *p* < 0.01, and *** *p* < 0.001.

We observed the morphological features of U87 MG brain cancer cells and astrocytes. As shown in Figure 2A,B, the growth of U87 MG cells was affected by soft plasma jet treatment, but astrocytes were not affected. We observed significant differences in growth between treated cells and untreated controls. The untreated U87 MG cells exhibited extensive spreading; the cell body, shape, and morphology were clearly visible. The treated cells showed a shrunken and rounded appearance. After plasma treatment, cancer cells started to detach from the surface, and many cells started to change from a spindle shape to a cobblestone-like morphology. Overall, plasma inhibited the growth of U87 MG cells, and plasma treatment for 180 s had the greatest inhibitory effect. The morphological modifications in the plasma-treated cells suggested damage to cellular metabolic processes. However, the morphology of astrocytes was clearly visible and was not substantially affected by plasma treatment. Astrocytes were not sensitive to soft plasma jet treatment, but U87 MG cells were highly sensitive.

### 2.3. U87 MG Cell Viability Decreases after Soft Plasma Jet Treatment

We investigated the ability of the soft plasma jet to selectively kill cancer cells. The cells were treated with soft plasma jets for various lengths of time, i.e., 30, 60, and 180 s, and were then incubated for 24 h along with an untreated group as the control. As shown in Figure 2C, in astrocytes (normal brain cells) treated with a soft plasma jet, the viability was nearly the same as that of the control cells. Figure 2D shows that, after 24 h of incubation, the viability of the U87 MG brain cancer cells treated with the soft plasma jet decreased. The viability was reduced in the plasma-treated U87 MG cells but not in the astrocytes.

### 2.4. Intracellular ROS and RNS Generation Induced by Soft Plasma Jet Treatment

ROS trigger the intrinsic apoptotic cascade by interactions with proteins in the mitochondrial porousness transition complex [36,37,38]. Mitochondrial depolarization results from oxidative stress produced by ROS. Atmospheric pressure plasma jets can generate ROS, thereby directly inducing apoptosis. As shown in Figure 2E,F, significantly higher levels of ROS and RNS fluorescence and relative amounts of ROS and RNS levels were observed following soft plasma jet treatment in U87 MG cells than in untreated controls.

### 2.5. Analysis of Plasma-Jet-Induced Apoptosis in U87 MG Cells

**Figure 3:**
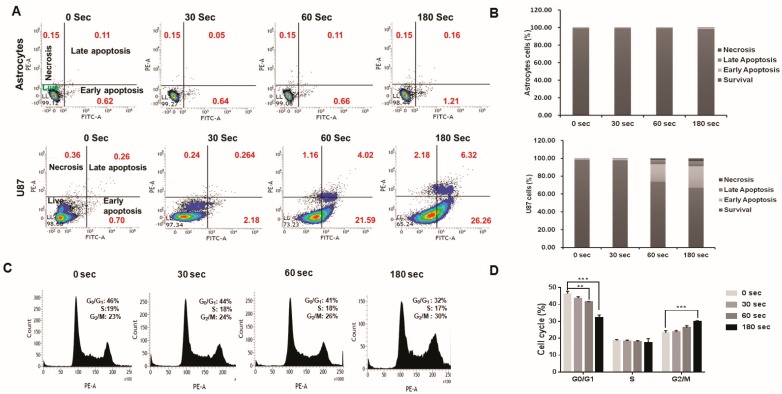
Apoptosis in astrocytes and U87 MG cells after plasma treatment: (**A**) representative flow cytometry (fluorescence-activated cell sorting (FACS)) dot plots of astrocytes and U87 MG cells prepared using the Annexin V-FITC Apoptosis Detection Kit; (**B**) summaries of frequencies of early and late apoptosis events for astrocytes and U87 MG cells; (**C**) effects of a soft plasma jet on the cell cycle distribution of U87 MG human brain cancer cells; (**D**) the percentage of cells in different cell cycle phases. The cells were treated for 30, 60, and 180 s and analyzed by flow cytometry. All values are presented as means ± SD of three independent experiments. ** *p* < 0.01, and *** *p* < 0.001.

To verify the proapoptotic impact, Annexin V-FITC and propidium iodide (PI) was used in U87 MG cells treated with a soft plasma jet. We assessed cell death for different treatment periods for cells exposed to a plasma jet. The plasma jet was used to treat cells for 30–180 s, which were then stained and analyzed as described above. Our results showed that soft plasma jet treatment resulted in time-dependent apoptosis. Significantly higher rates of early and late apoptosis were observed in the treatment group than in the control group (Figure 3A,B). Twenty-four hours after plasma treatment in U87 MG cells (lower panel), the total percentage of apoptotic cells in the control group was 0.96% (early: 0.70%; late: 0.26%), compared with 2.42% after the 30 s treatment (early: 2.18%; late: 0.24%), 25.61% after the 60 s treatment (early: 21.59%; late: 4.02%), and 32.58% after the 180 s treatment (early: 26.26%; late: 6.32%). Greater apoptotic effects were observed after treatment for 180 s. In contrast, the plasma treatment induced marginal necrosis (0.24%–2.18%). These data suggested that soft plasma jet treatment induces apoptotic cell death in U87 MG cells in a dose- and time-dependent manner.

The upper panels in Figure 3A,B showed that, 24 h after plasma treatment was applied to astrocytes, the total percentage of apoptotic cells in the control group was 0.73% (early: 0.62%; late: 0.11%) compared with 0.69% for the 30 s treatment (early: 0.64%; late: 0.05%), 0.77% for the 60 s treatment (early: 0.66%; late: 0.11%), and 1.37% for the 180 s treatment (early: 1.21%; late: 0.16%). Our findings indicated that astrocytes are not as sensitive to plasma treatment as U87 MG cells. We found no change in astrocyte apoptosis after plasma treatment.

### 2.6. U87 MG Cell Cycle Arrest after Plasma Treatment

Cell growth arrest initiated by stress or chemical compounds can be detected, when cells exhibit logarithmic growth rates and inducers are in balance with respect to the activation of repair mechanisms or apoptosis. We hypothesized that soft plasma jets influenced cell cycle regulation. Thus, cell cycle progression was evaluated in U87 MG cells seeded at 4 × 10^5^ cells/well incubated for 24 h. Cell cycle arrest was analyzed by fluorescence-activated cell sorting (FACS). Soft plasma jet treatment induced G_2_/M phase cell cycle arrest in U87 MG brain cancer cell lines. Treatment for 180 s or longer resulted in significant cell cycle arrest in the G_2_/M phase of U87 MG. After U87 MG cells were treated for 30, 60, and 180 s, we observed an obvious increase in cells in the G_2_/M phase. The results in Figure 3C,D revealed that, compared with that of untreated control cells, in which only 23% of cells were in the G_2_/M phase, after treatment for 30, 60, and 180 s, the percentages of cells in the G_2_/M phase increased significantly to 24%, 26%, and 30%, respectively. The flow cytometry revealed that soft plasma jet treatment induced G_2_/M cell cycle arrest. The cell cycle arrest in these cells led to an increase in cells in the G_2_/M phase with a corresponding reduction of cells in the G_0_/G_1_ and S phases and a significant increase in the population of G_2_/M phase cells.

### 2.7. Expression of Phosphorylated AKT (p-AKT) by Immunofluorescence

We evaluated p-AKT expression by immunofluorescence staining as shown in Supplementary Figure S1. AKT protein is well-known for controlling cell survival and proliferation inside cells. The inhibition of p-AKT induces apoptosis. p-AKT (observed as red fluorescence) was expressed in nuclei (observed as blue Hoechst 33342 staining). In control cells (cancerous), p-AKT expression increased. The confocal cell immunofluorescence revealed that the expression of p-AKT was high in control cells (cancerous cells). N-acetyl cysteine (NAC) was used as a broad antioxidant to investigate whether reactive species are involved in antitumor effects of plasma. In the group with the 180 s plasma treatment, the antitumor effect was rescued by NAC (Supplementary Figure S1). Plasma treatment suppressed p-AKT expression, but NAC inhibited this downregulation.

### 2.8. Plasma-Treated U87 MG Cells Induce Apoptosis through the p38 Mechanism

**Figure 4:**
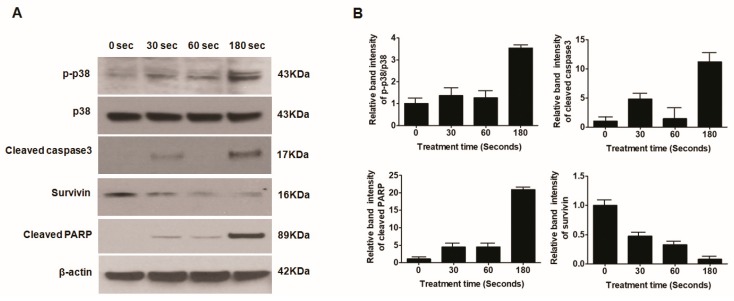
(**A**) Western blot analysis of protein expression in U87 MG cells; (**B**) relative band intensity as a function of treatment time.

To further confirm that apoptosis was induced by soft plasma jets, we performed a Western blot analysis of apoptosis-related proteins in U87 MG brain cancer cells. Plasma treatment induced apoptosis via DNA damage and apoptosis proteins like survivin, cleaved caspase b, and cleaved poly (ADP-ribose) polymerase (PARP). Our data illustrated that, with an increase in plasma dose, the expression levels of apoptosis-related proteins and proteins from the mitogen-activated protein kinase (MAPK) pathway, including p-p38, cleaved caspase-3, and PARP, increased significantly, as shown in Figure 4A,B.

The outcomes of our experiment confirmed that, after plasma treatment in U87 MG cells, caspase family proteins were activated, which could activate complicated intracellular apoptotic pathways and, finally, promote apoptosis. However, the precise signaling pathway is unknown but may be linked to the upregulation of p-p38 protein. More data are needed to identify signaling pathways, such as the mitochondria-related signaling pathway or death receptor signal transduction pathway, involved in the induction of apoptosis by plasma.

### 2.9. Plasma-Treated Cells Increase Survival Rate and Reduce Tumor Size in a U87 MG Mouse Model

**Figure 5:**
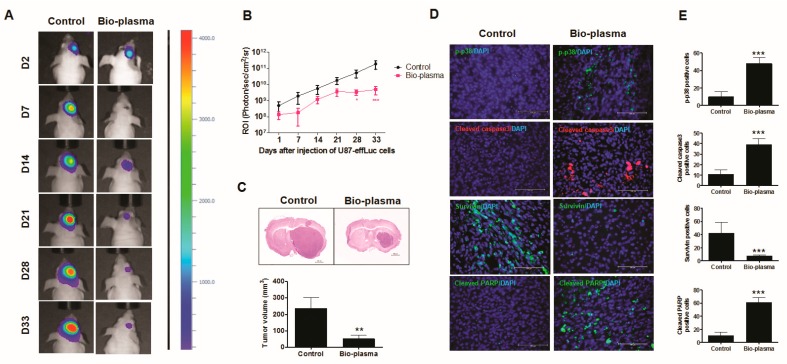
In vivo targeting of glioblastoma tumor with nonthermal atmospheric biocompatible plasma (NBP): (**A**) representative bioluminescence imaging; (**B**) region of interest (ROI) levels for changes in the tumor; (**C**) tumor size of the sectioned mouse brain by bioluminescence imaging and tumor volume; (**D**,**E**) expression of p-p38, cleaved caspase-3, cleaved poly (ADP-ribose) polymerase (PARP), and survivin determined via an immunofluorescence assay in mouse tumor tissues. All values are presented as means ± SD of three independent experiments. ** *p* < 0.01, and *** *p* < 0.001.

To confirm plasma treatment effects on U87 MG cells in an established animal model, bioluminescence imaging was performed to observe changes in tumor cell growth in vivo. The region of interest (ROI) was measured at a survival endpoint of day 44 postinjection. The ROI of the plasma-treated group was significantly reduced compared to that of the control, as shown in Figure 5A,B. Sectioned mouse brain tissues were used to accurately analyze the tumor size observed in bioluminescence imaging. The tumor volume of plasma-treated U87 MG cells was significantly lower (47.24 ± 16.94) than that of the control group (97.10 ± 11.78) (Figure 5C).

The immunofluorescence assay in mouse tumor tissues revealed that the expressions of p-p38, cleaved caspase-3, and cleaved PARP significantly increased in plasma-treated cells compared to those in the control and the expression levels of survivin decreased, as shown in Figure 5D,E. These results are consistent with those of the in vitro protein expression data.

## 3. Discussion

Since cellular apoptosis is promoted by plasma, the use of nonthermal atmospheric plasma has attracted substantial interest as a next-generation cancer therapy. Some research has proven that the impacts of plasma are noticeably selective for cancer cells [39,40,41,42,43]. Time-dependent apoptosis has been confirmed in plasma-treated cancer cells, with no differences between plasma-treated normal cells and untreated cells [42,44]. In plasma-based medicine, various approaches, such as plasma jets, corona discharge, and dielectric barrier discharge (DBD), have been widely studied in vitro and in vivo [42,45]. These types of plasma can be directly applied to skin cancer cells, but they are not applicable for further systemic cancer treatment. Plasma jets have deeper penetration than DBD plasma, which mostly generates surface discharge [46]. Although brain tumors are generally difficult to directly reach using plasma devices, plasma has shown potential for brain cancer therapy. In this study, we used a unique jet-type plasma device for the treatment of brain tumors in vitro. Cell toxicity, apoptotic effects, and mechanisms underlying the effects of plasma were examined.

We observed that NBP had selective apoptotic effects in brain cancer cells. Plasma treatment resulted in a greater decrease in cell viability in U87 MG cells than in astrocytes. U87 MG cells reacted more sensitively to NBP than normal cells. NBP-J was shown to affect cancer cell morphology and induce apoptosis, while leaving normal cells relatively unharmed. NBP can generate ROS and RNS, which may have key mechanistic functions in cancer therapy [47].

In the current study, we found that plasma treatment in vitro increased the rate of apoptosis, related to cell cycle arrest at the G_2_/M phase. Cell cycle control is one of the main regulatory mechanisms underlying cell growth [48,49,50,51]. Our flow cytometry analyses showed that the percentage of G_2_/M cells increased dose dependently, thus preventing the generation of daughter cells.

We assessed apoptosis-related proteins by Western blotting to evaluate the precise molecular mechanism, through which plasma treatment causes cell cycle arrest and apoptosis. We found that the expression levels of apoptosis proteins increased in response to plasma treatment. Our findings also indicated that soft plasma jets resulted in ROS and RNS generation, thereby inducing an apoptotic signaling cascade by activating p38/MAPK signaling, which further led to the actuation of PARP and cleaved caspase-3 as apoptosis proteins. The most complicated intracellular signal transduction structures are present in the MAPK signaling pathway and, in common, are activated using an extensive cluster of intracellular or extracellular stimuli [52]. Three common components of the MAPK signaling pathway are ERK, JNK, and p38 MAPK pathways, frequently participating in genetic processes despite crosstalk between these signaling pathways at the upper levels of cascades [53]. In this study, we observed ROS uptake in U87 MG cancer cells treated with NBP-J.

Plasma treatment was toxic to U87 MG cells but not to astrocytes. Generally, higher intracellular ROS concentrations are present in cancer cells than in normal cells, and hence they face more challenges in managing additional oxidative damage due to RONS from plasma, whereas healthy cells can protect themselves more easily, diminishing oxidative pressure and restoring stability. We demonstrated that NBP-J has the potential to selectively induce apoptosis in brain cancer cells via the generation of ROS and RNS. Intracellular ROS and RNS can play key roles in cancer cell apoptosis. This tumor inhibition is mediated by cell cycle arrest, and the duration of treatment is important for determining the levels of cytotoxicity and apoptosis. Overall, plasma-treatment-induced cell death, morphological changes, and cell cycle arrest increased the expression of genes related to apoptosis (p-p38, caspase-3, cleaved *PARP*, and survivin) in the U87 MG cancer cell line. Our in vivo study suggested that the addition of plasma-treated U87 MG cells reduced the tumor volume.

Although more studies are required to determine the exact mechanisms, these results indicated the anticancer activity of soft-jet plasma, providing new insights into molecular mechanisms. Atmospheric plasma is administered in the open air and at room temperature; therefore, this device for cancer treatment has clinical value, particularly given the potential to target cancerous cells over healthy cells. It could also be applied within postoperative healing processes. Our results provide a fundamental basis for the development of a clinically suitable device, e.g., a needle-type plasma jet, to be directly applied to the brain. The main goal of this work was a preclinical proof-of-concept for plasma treatment in brain cancer; an indication of preclinical models for plasma treatment will be useful for further study. However, in the future, we will perform in vivo work by injecting U87 cells as the naïve condition in the mouse brain, then allowing the tumor to form and then apply direct NBP-J treatment. Additionally, we will develop a protocol for the clinical application of a needle-type plasma jet, which is our next focus.

## 4. Materials and Methods

### 4.1. Experimental Plasma Device and Measurement of Physical Properties

Figure 1A depicts a schematic of an atmospheric soft plasma jet system and an air gas flow. The system included a high-voltage inner electrode and a grounded (outer) electrode in stainless steel. The inner electrode was composed of stainless steel with a size of 1.2 mm × 0.2 mm, and the outer electrode was prepared by stainless steel with a length of 6 mm, a thickness of 0.2 mm, and a 0.7 mm diameter hole for the generation of plasma. Air was used as the feeding gas, and the flow speed was controlled using an analog controller. A charge-coupled spectrometer (HR400; Ocean Optics, Largo, FL, USA) was used to measure optical emission spectroscopy. The light intensity was recorded under conditions of wavelengths emitted from the device.

### 4.2. Cell Culture

U87 MG (human brain cancer cell line) and normal human astrocytes (NHA) were purchased from the Korean Cell Line Bank (KCLB, Seoul, Korea) and Lonza (CC-2565, Basel, Switzerland), respectively. U87 MG (human brain cancer cell line) and astrocytes were cultured DMEM (Life Technologies, Carlsbad, CA, USA) added with 10% FBS (Gibco, Grand Island, NY, USA) and 1% antibiotic (penicillin/streptomycin; Gibco, Grand Island, NY, USA). A humidified incubator with 5% CO2 at 37 °C was used to maintain the cultured cells. All experiments were performed using cells in the exponential phase. The cell suspensions were seeded on 100 mm cell culture plates and incubated for around 20–24 h to reach confluence prior to experiments. A cell viability (Alamar Blue, Invitrogen, CA, USA) assay, analysis of cell morphology, intracellular ROS and RNS detection, cell cycle arrest assay, immunofluorescence assay, and Western blotting were used to evaluate the effects of NBP-J. NAC (5 mM) was applied as an ROS scavenger.

### 4.3. Cytotoxicity Assay

The cytotoxic effect of NBP-J was monitored using Alamar Blue, a redox fluorogenic sign of metabolic reduction (Invitrogen, Thermo Fisher Scientific, Waltham, MA, USA). Cells were seeded at a concentration of 1 × 10^5^ cells/well into 24-well plates (Falcon, BD Biosciences, San Jose, CA, USA) and incubated for 1 day to allow for cell adhesion and stability. The medium was replaced, and cells were treated with NBP-J for 30, 60, and 180 s followed by an additional incubation period. A control group with no plasma treatment was included in all assays. After 24 h of incubation, the medium was changed to Alamar Blue mixed with DMEM in a 1:10 ratio. Alamar Blue reagent is sensitive to light; thus, plates were covered with aluminum foil and incubated for 3 h. After 3 h, 100 µL of the medium was added to a 96-well black plate. Reactions were monitored using a microplate reader (Biotek, Winooski, VT, USA) with an excitation wavelength range of 570–560 nm and an emission wavelength of 590 nm. For all assays, three independent sets of tests were performed.

### 4.4. Intracellular ROS and RNS Detection

U87 MG cells were seeded on 12-well plates with round glass coverslips at 5 × 10^4^ cells per dish. After 24 h, cells were treated for 30, 60, and 180 s with a soft plasma jet followed by incubation for 24 h. An ROS and RNS detection kit (Invitrogen, CA, USA) was used to assess intracellular ROS and RNS levels following the manufacturer’s instructions. 2,7-Dichlorodihydrofluorescein diacetate (H2DCF DA; Invitrogen, CA, USA) was used to oxidize cells to release intense green fluorescence using intracellular ROS. For RNS detection, DAF-FM was used. Laser scanning confocal microscopy was used to obtain fluorescence images at a 20× magnification.

### 4.5. Apoptosis Assay

To detect apoptosis, Annexin V-FITC (fluorescein isothiocyanate) and PI staining were performed, and cells were evaluated using a FACSVerse cytometer (BD, San Jose, CA, USA) and an Annexin V-FITC Apoptosis Detection Kit (BD, San Jose, CA, USA) following the manufacturer’s protocol. In short, cells were treated with plasma and incubated for 24 h, after which they were collected, washed with Dulbecco’s phosphate-buffered saline (DPBS), stained with Annexin V-FITC and PI and analyzed using flow cytometry. FACSVerse software (BD Bioscience, San Jose, CA, USA) was used to analyze the percentage of cells in four populations, including FITC^−^/PI^−^ (living cells), FITC^+^/PI^−^ (early apoptotic cells), FITC^+^/PI^−^ (late apoptotic cells), and FITC^−^/PI^+^ (necrotic cells).

### 4.6. Cell Cycle Flow Cytometry Analysis

To evaluate the effect of soft plasma jets on cell cycle progression, U87 MG cells were seeded at 4 × 10^5^ cells per well and incubated for 24 h. Cell cycle arrest was analyzed by FACS. After 24 h of treatment, cancer cells were harvested, fixed with 75% ice-cold ethanol for 24 h, treated with RNase-2 (Sigma-Aldrich, St. Louis, MO, USA), stained with PI, and analyzed by flow cytometry.

### 4.7. Immunofluorescence Staining for p-AKT

To investigate the regulation of the p-AKT expression in U87 MG cells, immunofluorescence staining was performed. Discussion related to immunofluorescence staining for p-AKT is provided in Supplementary Figure S1.

### 4.8. Western Blot Analysis

For protein extraction, cells were treated with plasma at different time points (30, 60, and 180 s compared with the control) and harvested by a radioimmunoprecipitation assay (RIPA) buffer after 24 h of culturing. For immunoblot confirmation, 20 µg of protein was used, and the analysis was performed as previously described. Primary antibodies were used against p38 and its phospho form (Thr180/Tyr182, Cell signaling, Danvers, MA, USA), cleaved caspase-3 (Cell Signaling, Danvers, MA, USA), survivin, and cleaved PARP (Abcam, Cambridge, United Kingdom), and GAPDH (Sigma-Aldrich, St. Louis, MO, USA). The intensity of bands was detected using ImageJ software. Data were normalized according to their corresponding β-actin levels.

### 4.9. Orthotopic U87 MG Xenograft Mouse Model

All procedures involving mice in tests were approved by the Institutional Animal Care and Use Committee (IACUC number: KWU-PBRC1905001) at Kwangwoon University (Seoul, Korea). Seven-week-old female BALB/c-nude mice were anesthetized by intraperitoneal (i.p.) injection of 30 mg/kg zoletil (Virbac SA, France) and 10 mg/kg xylazine (Bayer Inc., Toronto, Ontario). As previously described, luciferase-expressing U87 MG (U87 MG-effluc) was used for bioluminescence imaging [54]. The cells were expanded to 90% confluence and divided into two groups. The first group was harvested directly in PBS (Phosphate Buffered Saline) and acted as a control, whereas the second group was treated with NBP-J for 3 min and extracted to be injected (1.2 × 10^6^ in 3 µL) with a stereotaxic device using a Hamilton syringe at an injection rate of 1 µL/min into the brains. Stereotaxic coordinates were selected to be 1 mm anterior and 2 mm lateral to the bregma and 3 mm deep from the dura.

### 4.10. Bioluminescence Imaging and Survival Analysis

Brain tumor growth was observed via bioluminescence imaging using VISQUE™ In Vivo Elite Imaging System followed by imaging every alternate day until day 44 postinjection, after which the mice were euthanized. For the detection of in vivo live imaging, the mice were i.p. administered 150 mg/kg D-Luciferin (Caliper Life sciences, Hopkinton, MA, UA). After anesthetizing the mice with 2% isoflurane (Piramal Healthcare, Bethlehem, PA, UA) in 100% O_2_, pictures were captured by recording the bioluminescent indication for 8 min. The signals were analyzed and quantified by calculating the luminescent intensity in the ROI using Clavue software, registered with the imaging system.

### 4.11. Tumor Volume and Immunofluorescence Staining

For histological analysis, the mice were sacrificed after 44 days of the U87 MG efflux cell injection. Cardiac perfusion was performed and frozen blocks were prepared for sectioning as previously reported [54]. The tissues were stained with hematoxylin and eosin (H&E) to mark the tumor volume within the sections. Immunofluorescence was performed using primary antibodies as follows: p-p38 (1:400, Cell Signaling), cleaved caspase-3 (1:100, Abcam), survivin (1:250, Abcam), and cleaved PARP (1:400, Abcam). The secondary antibody used was Alexa Fluor 594- or 488-conjugated with anti-IgG or 594-conjugated antigoat IgG (1:500; Invitrogen, Carlsbad, CA, USA), and the sections were mounted with anti-fading solution containing 4′-6-diamidino-2-phenylindole (DAPI, Vector Laboratories, Burlingame, CA, USA). Fluorescent images were obtained using a fluorescence microscope (Leica, DMi8, Wetzlar, Germany). Positively stained slides were quantified from a minimum of three randomly stained slides.

### 4.12. Statistical Analysis

Results are presented as a percentage of controls ± SD or means ± SD. Student’s *t*-tests were used to analyze statistical differences between two groups, and multiple groups were surveyed using one-way analysis of variance (ANOVA) followed by a post-hoc test. The survival data are presented in Kaplan–Meier survival graphs and were analyzed by the log-rank test. Statistical analyses were conducted using GraphPad Prism software (La Jolla, CA, USA), and each experiment was performed independently at least 3 times. Differences with values of *p* < 0.05 were considered statistically significant.

## 5. Conclusions

In conclusion, our results indicated that plasma treatment has an anticancer effect on U87 MG cells in both in vitro and in vivo by activating the cellular apoptosis mechanism via the p38/MAPK mechanism. The plasma-activated proteins like cleaved PARP and cleaved caspase-3, leading to increased tumor cell death. We think that these results provide important insight into the molecular mechanism underlying apoptosis in brain cancer cells. Although more studies are required to determine the detailed mechanisms, our results suggested that soft plasma jets can be used as a potential therapeutic approach for brain cancer.

## Supplementary Materials

The following are available online at https://www.mdpi.com/2072-6694/12/1/245/s1, Figure S1: Immunofluorescence-based visualization of p-AKT expression with plasma treatment and NAC.
