# Supplementary material for: Non-Thermal Atmospheric Pressure Bio-Compatible Plasma Stimulates Apoptosis via p38/MAPK Mechanism in U87 Malignant Glioblastoma
Source: Cancers (Basel). 2020 Jan 19;12(1):245. doi: 10.3390/cancers12010245 (PMC7016658; doi:10.3390/cancers12010245)
Supplement: Supplementary file 1 [file cancers-12-00245-s001.pdf]

Non-thermal Atmospheric Pressure Bio-Compatible Plasma Stimulates Apoptosis via p38/MAPK Mechanism in U87 Malignant Glioblastoma

Mahmuda Akter, Anshika Jangra, Seung Ah Choi, Eun Ha Choi and Ihn Han

Running title: Soft jet plasma induction of apoptosis in U87 MG brain cancer cell line.

Materials and Methods

S1: Immunofluorescence Staining for p-AKT

To investigate the regulation of the p-AKT expression in the U87 MG cells immunofluorescence staining was performed. Briefly, U87 MG cells were cultured on glass coverslips at the base of 12-well plates and incubated for 24 hours. Cells were fixed for 20 min with 4% paraformaldehyde and permeabilized in PBS containing 0.1–0.25% Triton X-100. To block nonspecific binding, cells were incubated with 10% BSA in TBS with 0.1% Tween 20 for one hour. After blocking non-specific staining, the cells were incubated with the primary antibody (Cell signaling, 1:1000, Danvers, Massachusetts, USA) which shows phosphorylation at Ser473) in a humidified chamber at 4°C overnight. After three washes with PBS, the secondary antibody (Goat polyclonal secondary antibody to mouse IgG - H&L; Alexa Fluor® 594, BioRad, 1:5000, California, USA) was added and cells were incubated for 1 hour at room temperature in the dark. Cells were then stained with Hoechst 33258 for 15 minutes to identify nuclei. Images were captured using a confocal fluorescence microscope.

Results

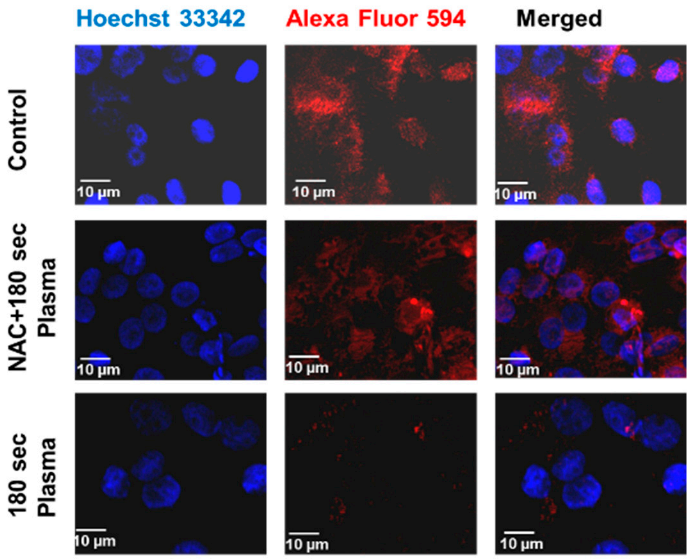

Figure S1. Immunofluorescence-based visualization of p-AKT expression with plasma treatment and NAC. Red: fluorescence signal indicating marker expression. Blue: Nuclei visualized by Hoechst 33342 dyes NAC showing scavenges ROS, thereby affecting the anti-tumor effect of the soft plasma jet.
